# Preparation and characterization of 2-hydroxyethyl starch microparticles for co-delivery of multiple bioactive agents

**DOI:** 10.1080/10717544.2021.1955043

**Published:** 2021-07-21

**Authors:** Sreekanth Reddy Obireddy, Wing-Fu Lai

**Affiliations:** a Department of Chemistry, Sri Krishnadevaraya University, Anantapur, India; b Shenzhen Key Laboratory of Steroid Drug Discovery and Development, School of Life and Health Sciences, The Chinese University of Hong Kong (Shenzhen), Shenzhen, China; c Ciechanover Institute of Precision and Regenerative Medicine, The Chinese University of Hong Kong (Shenzhen), Shenzhen, China; d School of Education, University of Bristol, Bristol, UK

**Keywords:** 2-Hydroxyethyl starch, sustained release, co-delivery, ofloxacin, ketoprofen, microparticles, antibacterial activity

## Abstract

The present study reports the generation of 2-hydroxyethyl starch microparticles for co-delivery and controlled release of multiple agents. The obtained microparticles are characterized by using Fourier transform infrared spectroscopy, differential scanning calorimetry, X-ray diffraction analysis, energy-dispersive X-ray spectroscopy, and scanning electron microscopy. By using ofloxacin and ketoprofen as drug models, the release sustainability of the microparticles is examined at pH 1.2, 5.4, and 6.8 at 37 °C, with Fickian diffusion being found to be the major mechanism controlling the kinetics of drug release. Upon being loaded with the drug models, the microparticles show high efficiency in acting against *Escherichia coli* and *Bacillus cereus*. The results suggest that our reported microparticles warrant further development for applications in which co-administration of multiple bioactive agents is required.

## 1. Introduction

**Figure 1.**
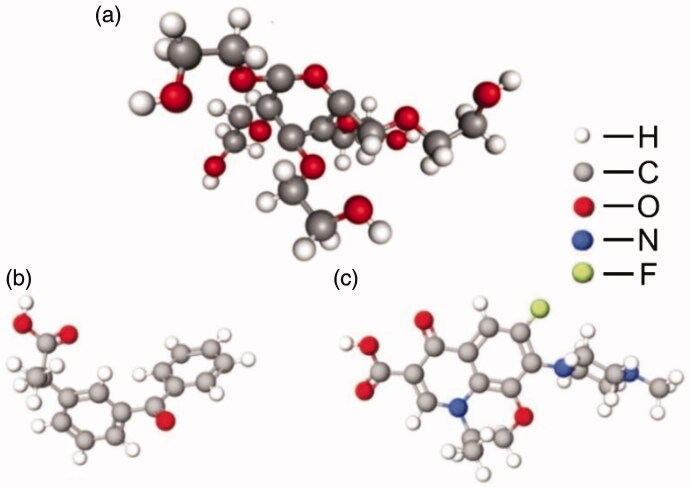
Structures of (a) HES, (b) KP, and (c) OFLX.

Bioactive agent carriers (such as hydrogels, microparticles, and nanoparticles) are generally produced from polymeric materials (Ali & Ahmed, 2018; Aoki & Saito, 2020). Compared with synthetic polymers, natural polymers are generally more biodegradable and less toxic (Lai & Lin, 2009; Lai, 2014; Lai & Wong, 2018; Lai, 2020). Till now a large variety of carriers have already been developed and reported in the literature (Lee et al., 1999; Wei et al., 2009; Fan et al., 2016); however, most of them are designed for single-drug delivery (Lai & Shum, 2015, 2016; Lai et al., 2019; Reddy et al., 2021; Sreekanth Reddy et al., 2021). They either fail to be loaded with multiple agents effectively (Lai & Rogach, 2017) or lack the capacity of enabling the release rate of each of the co-delivered agents to be precisely controlled (Lai et al., 2016). This limits the applications of these carriers in multi-drug therapy. The objective of this study is to develop a carrier to fill this technical gap to make the co-administration of multiple agents possible. To achieve this goal, hydroxyethyl starch (HES) is adopted (Figure 1). It is an FDA-approved polymer derived from starch, which is well-known to be cheap, highly biodegradable, and highly biocompatible (Narayanan et al., 2015). Although native starch has also been used for the generation of carriers in the form of hydrogels (Van Nieuwenhove et al., 2016, 2017), microspheres (Pereira et al., 2013), and nanocomposites (Bakrudeen et al., 2016), many of these carriers degrade rapidly *in vivo* and show poor drug release sustainability (Fang et al., 2008). Comparing with native starch, HES is more resistant to degradation (Michailova et al., 2001). It has been widely used for volume therapy (Nanaki et al., 2012), and degrades steadily in the presence of serum α-amylase (Sakr et al., 2007; Baier et al., 2012).

In bioactive agent delivery, HES shows a number of favorable features (Treib et al., 1999; Noga et al., 2012). For instance, HES is non-immunogenic, and has an extended retention time in vascular compartments, resulting in a controllable degradation profile in the presence of serum α-amylase. Due to hydroxyethylation, it also shows higher aqueous solubility than native starch and possesses excellent protein repellent properties. These unique features make HES a very promising material for biomedical applications. In this study, we use HES to generate, via the use of a sonication method, microparticles (MPs) for co-delivery of ketoprofen (KP) and ofloxacin (OFLX). KP is an effective non-steroidal anti-inflammatory drug widely used for tackling inflammation in osteoarthritis and rheumatoid arthritis (Rhee et al., 2001; Arida & Al-Tabakha, 2007; Manikkath et al., 2017); whereas OFLX is a synthetic quinolone/fluoroquinolone antibiotic widely used to treat infections in urinary, gastrointestinal and respiratory tracts (Cheng et al., 2002; Zivanovic et al., 2006). Both of them have a long track record of clinical use. By using these two drugs as models, the generated drug-loaded MPs are characterized by using diverse techniques to examine the size, shape, morphology, drug release kinetics, and bacterial properties as carriers of multiple bioactive agents.

## 2. Experimental

### 2.1. Materials

HES, OFLX, KP, and pentasodium tripolyphosphate (STPP) were purchased from Sigma-Aldrich (St. Louis, MO, USA). Absolute ethanol and sodium hydroxide were purchased from Sd. Fine chemicals (Mumbai, India). Millipore water was used throughout the study.

### 2.2. Preparation of HES MPs

HES MPs were prepared by using a sonication method. In brief, the HES solution was prepared by adding 0.4 g of HES to 10 mL of millipore water and was agitated at 60 °C for 1 h. After that, the solution was cooled to room temperature, followed by the addition of a 0.1 N sodium hydroxide solution until the pH of the solution reached 5.5. After adding appropriate amounts of OFLX and KP, the solution was stirred at 300 rpm until a homogenous dispersion was attained. STPP was added. The solution was stirred at 300 rpm for another 1 h and was then sonicated for 10 min. After that, it was mixed with absolute ethanol, stirred at 2000 rpm for 5 min, and then sonicated using a probe-type sonicator at 37 °C for 15 min at 4 s pulse on/off mode. The generated MPs were collected, washed twice with double distilled water, frozen, and dried. The compositions of different formulations of MPs were shown in Table 1.

**Table 1.** Compositions of different formulations of MPs and the corresponding encapsulation efficiency.

Formulation	HES (% w/v)	STPP (% w/w)	OFLX (% w/w)	KP (% w/w)	EE (%)	
					OFLX	KP
HES-1 MPs	4	5	25	0	42.2 ± 0.016	N/A
HES-2 MPs	4	5	40	0	46.2 ± 0.039	N/A
HES-3 MPs	4	5	50	0	47.6 ± 0.021	N/A
HES-4 MPs	4	10	25	12.5	41.3 ± 0.022	54.4 ± 0.016
HES-5 MPs	4	15	25	12.5	40.2 ± 0.011	50.3 ± 0.016
HES MPs	4	5	0	0	N/A	N/A

Abbreviation: EE, encapsulation efficiency; N/A, not applicable.

### 2.3. Structural characterization

A Fourier transform infrared (FTIR) spectrometer (MB-3000; ABB Bomem, Quebec, Canada) was used to record the FTIR spectra of OFLX, KP, HES MPs, and drug-loaded MPs. The spectra were analyzed using the Horizon MB^™^ FTIR software. Differential scanning calorimetry (DSC) thermograms of OFLX, KP, HES MPs, and drug-loaded MPs were recorded from 30 °C to 350 °C at a heating rate of 10 °C/min under a nitrogen atmosphere using a simultaneous thermal analysis (STA) instrument (SDT Q600; TA Instruments, New Castle, PA, USA). Powder X-ray diffraction (XRD) patterns of OFLX, KP, HES MPs, and drug-loaded MPs were obtained using an X-ray diffractometer (X’Pert PRO, PANalytical Company, Almelo, Netherlands) with CuKα radiation (*λ* = 1.54060 Å) at a counting rate of 5°/min. The compositions and morphological features of MPs were analyzed using scanning electron microscopy (SEM) (JSM 840 A; JOEL Ltd., Peabody, MA, US) equipped with energy-dispersive X-ray spectroscopy (EDS).

### 2.4. Determination of EE

10 mg of HES MPs was dispersed in 10 mL of phosphate-buffered saline (PBS) [containing 5% (v/v) ethanol]. After that, the MPs were sonicated for 10 min and were crushed to extract the loaded drugs from the MPs. Using an ultraviolet-visible (UV-Vis) spectrophotometer, the concentration of OFLX was determined at the *λ*_max_ of 287.6 nm and that of KP was measured at the *λ*_max_ of 254.7 nm. EE was calculated using the following equation as previously reported (Obireddy & Lai, 2021).

(1) $$\mathrm{EE}(\%)=\frac{w_{f}}{w_{d}}\times 100\%$$

where w*_f_* was the mass of the unloaded drug, and w*_d_* was the total weight of the drug added during the process of drug loading.

### 2.5. Evaluation of drug release kinetics

Profiles of drug release were determined at 37 °C using a dissolution tester (DS8000; Lab India, Mumbai, India). 100 mg of drug-loaded MPs was packed in a dialysis bag. The bag was then suspended in 500 mL of PBS at different pH values (1.2, 5.4, and 6.8) at 37 °C. At pre-determined time intervals, 3 mL of the release medium was withdrawn and was replenished by the same amount of PBS. Using a UV-Vis spectrophotometer, the concentration of OFLX was determined at the *λ*_max_ of 287.6 nm and that of KP was measured at the *λ*_max_ of 254.7 nm. To determine the release kinetics of MPs, the release curves were fitted into different kinetic models (including the zero-order model, the first-order model, the Higuchi model, and the Korsmeyer–Peppas model) (Costa & Sousa Lobo, 2001; Venkata Prasad et al., 2012; Dozie-Nwachukwu et al., 2017). Based on the correlation coefficients, the best drug release kinetics model was identified (Arhewoh & Okhamafe, 2004).

### 2.6. Evaluation of antibacterial activity

The antibacterial activity of OFLX-loaded MPs was examined by using the agar well diffusion technique. In brief, 100 µL of *Escherichia coli* (MTCC-1668) or *Bacillus cereus* (MTCC-4079) was dispersed on Luria–Bertani (LB) plates. Three wells were created on each plate. Aqueous solutions of test samples (HES-1 MPs, HES-2 MPs, and HES-3 MPs) were prepared at a concentration of 1 mg/mL. 100 µL of a test sample was added to each well. The plates were incubated at 37 °C for 24 h. After that, zones of inhibition were determined as previously described (Marslin et al., 2015).

## 3. Results and discussion

### 3.1. Structural characterization of MPs

**Figure 2.**
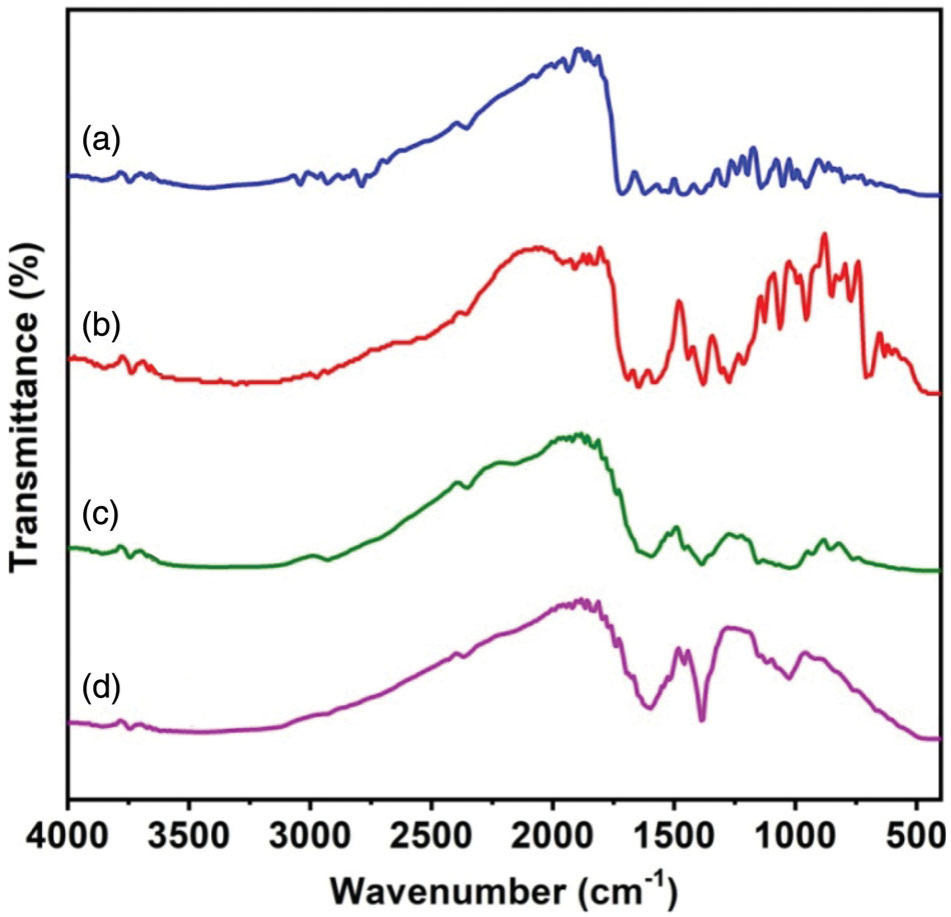
FTIR spectra of (a) OFLX, (b) KP, (c) HES MPs, and (d) HES-4 MPs.

Structures of OFLX, KP, HES MPs, and HES-4 MPs are examined by using FTIR spectroscopy (Figure 2). The spectrum of OFLX shows a characteristic peak at 3418 cm^−1^ which is attributed to O–H stretching vibrations. Peaks at 1720, 1384, and 1142 cm^−1^ are assigned to C=O stretching vibrations, C–O stretching vibrations, and C–N stretching vibrations, respectively (Al-Omar, 2009; Vashisth et al., 2017). The spectrum of KP shows a characteristic peak at 3384 cm^−1^, which is caused by O–H stretching vibrations. Peaks at 1693 and 1647 cm^−1^ are contributed by C=O stretching vibrations of the COOH group and the keto group, respectively. The peak at 1381 cm^−1^ is due to C–O stretching vibrations (Manikkath et al., 2017).

The spectrum of HES MPs shows characteristic peaks at 3407 cm^−1^ and 1596 cm^−1^, which are attributed to O–H stretching vibrations and in-plane vibrations of the O–H group, respectively. Peaks at 1380 and 1018 cm^−1^ are assigned to C–O stretching vibrations (Silva et al., 2014). The peaks found in the spectrum of HES MPs are also observed in the spectrum of HES-4 MPs; however, the peak at 1596 cm^−1^ in the spectrum of HES MPs is shifted to 1600 cm^−1^ in the spectrum of HES-4 MPs. This is caused by physical interactions between HES and loaded drugs. In addition, peaks at 1458 and 1116 cm^−1^ are assigned to C–F stretching vibrations. The presence of these peaks further confirms that no chemical interaction takes place between the loaded drugs and HES. The interactions between the loaded drugs and HES are physical in nature (Li et al., 2014).

**Figure 3.**
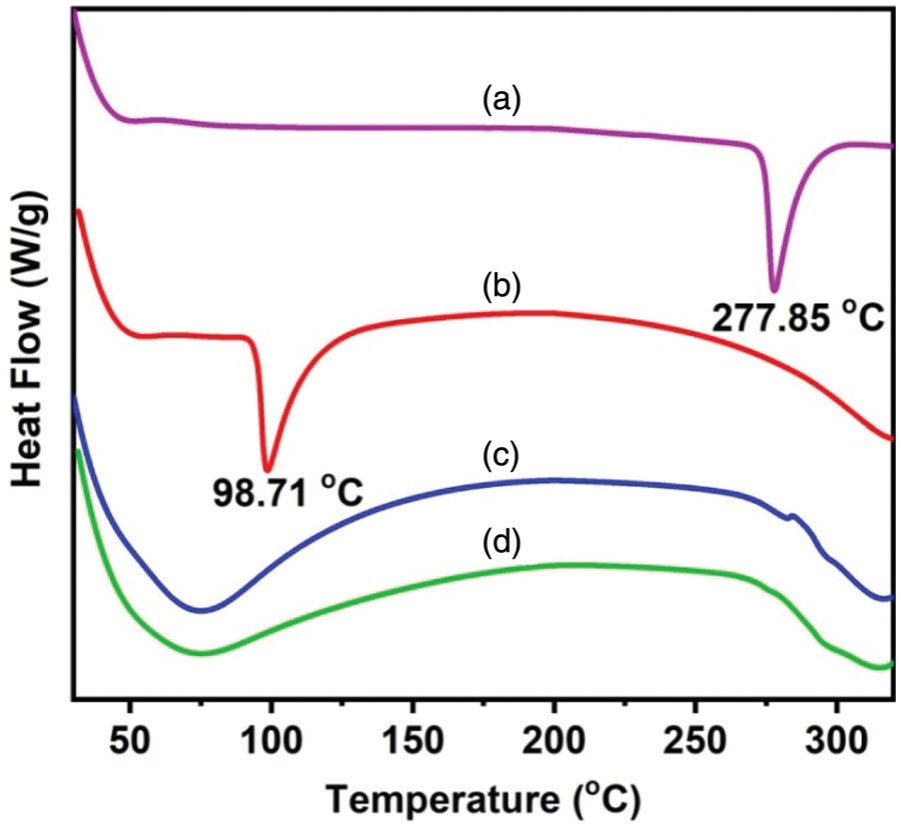
DSC thermograms of (a) OFLX, (b) KP, (c) HES-4 MPs, and (d) HES MPs.

**Figure 4.**
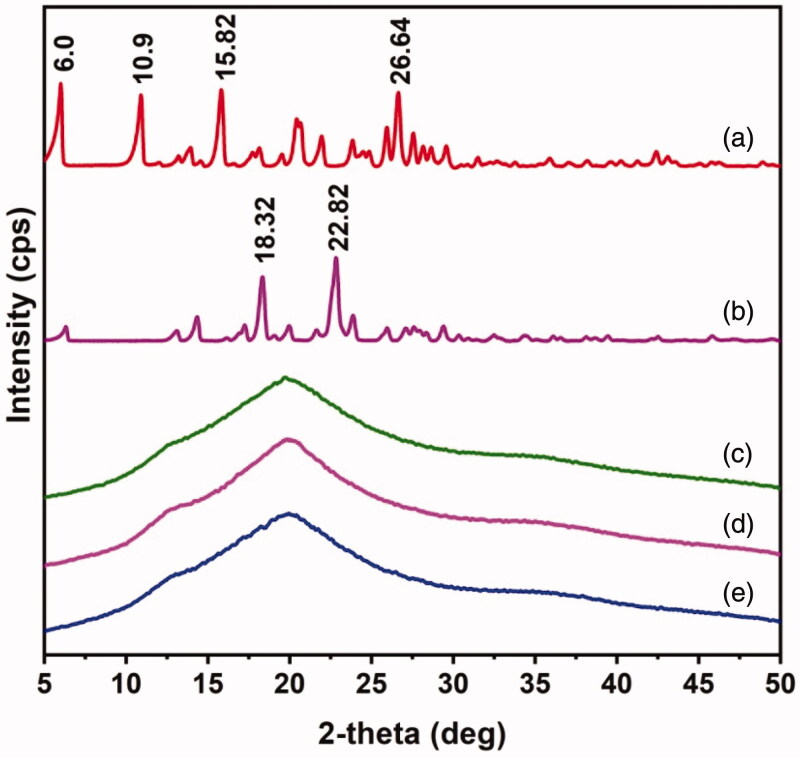
Powder XRD patterns of (a) OFLX, (b) KP, (c) HES-4 MPs, (d) HES-3 MPs, and (e) HES MPs.

The dispersion of drugs inside the polymeric matrix of MPs is examined by using DSC and XRD analyses. DSC thermograms of OFLX, KP, HES MPs, and HES-4 MPs are shown in Figure 3. OFLX and KP show sharp peaks at 277.85 °C and 98.71 °C, respectively. These peaks correspond to the melting points of the drugs. They are not found in HES-4 MPs. This confirms that the drug molecules are uniformly dispersed in the polymeric matrix of the MPs. XRD patterns of OFLX, KP, HES-3 MPs, HES-4 MPs, and HES MPs are shown in Figure 4. Intensive peaks are observed at 2*θ* of 10.9°, 15.82° and 26.64° in the XRD pattern of OFLX; whereas peaks at 2*θ* of 18.32° and 22.82° are detected in the XRD pattern of KP. These peaks are contributed by the crystalline structures of the drugs. These peaks are absent in the XRD patterns of HSE-3 and HSE-4 MPs, indicating that both OFLX and KP are uniformly dispersed in the polymeric matrix of the MPs. A similar observation has been made in the XRD pattern of rosuvastatin-loaded β-cyclodextrin-based hydrogel microparticles reported by Ahmad and coworkers (Arhewoh & Okhamafe, 2004).

### 3.2. Microscopic and compositional analyses

**Figure 5.**
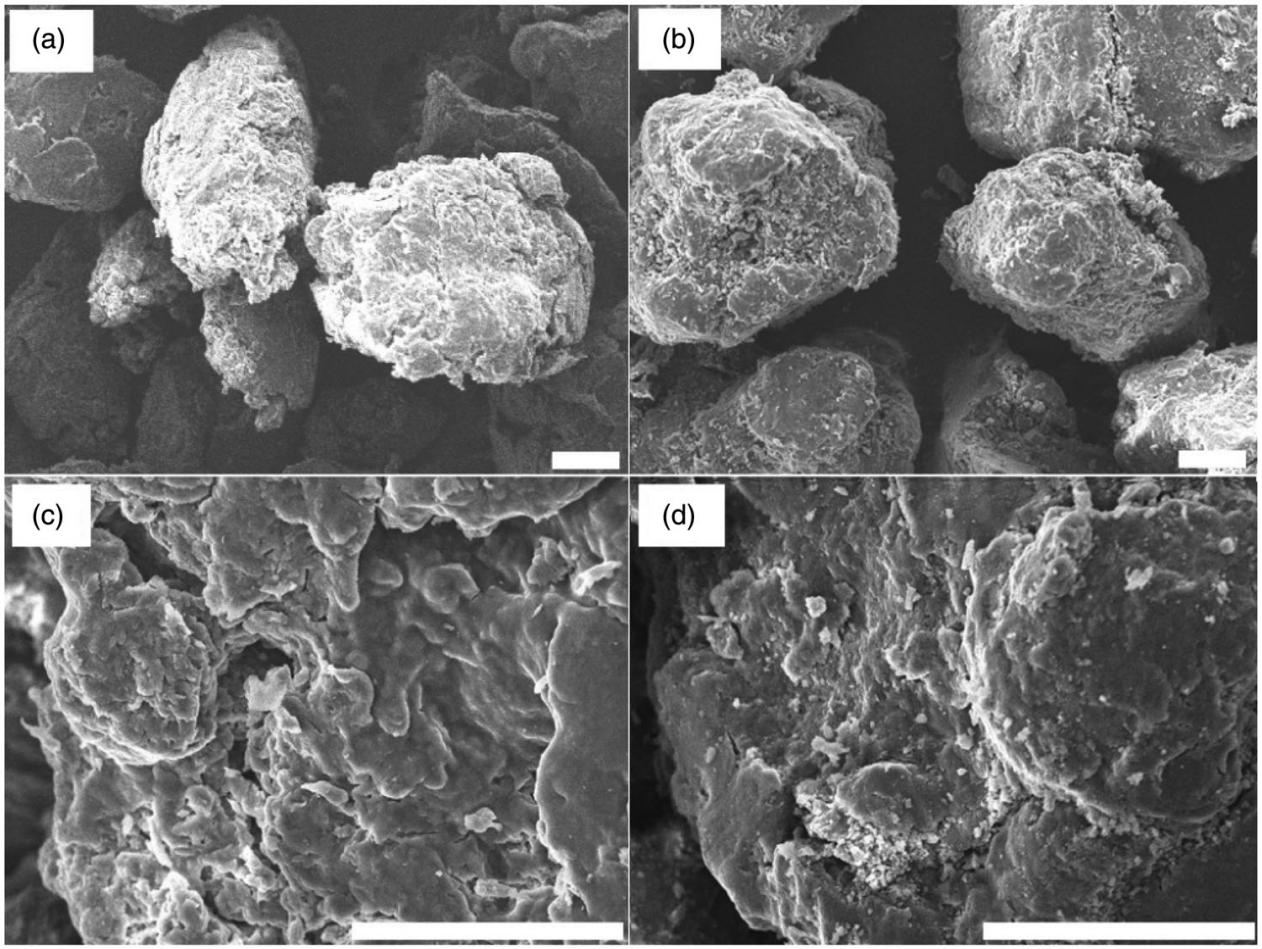
SEM micrographs of (a, c) HES MPs and (b, d) HES-4 MPs. Scale bar = 100 µm.

**Figure 6.**
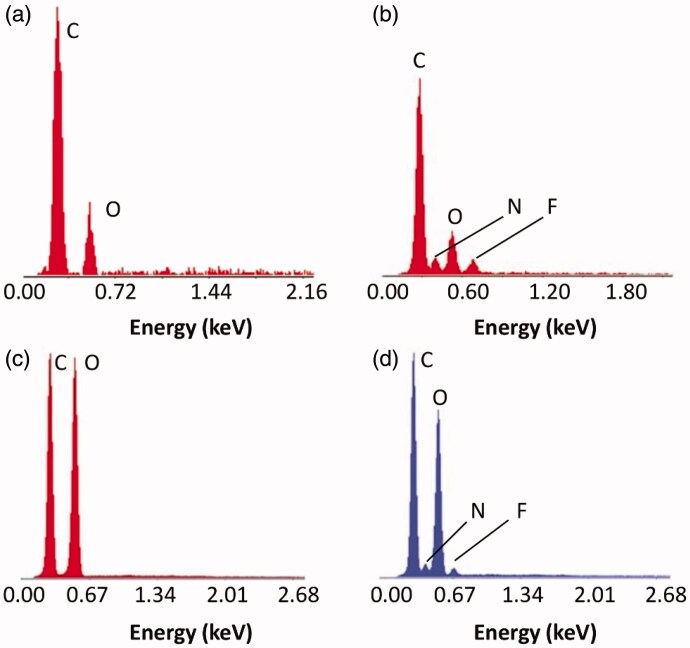
EDS spectra of (a) KP, (b) OFLX, (c) HES MPs, and (d) HES-4 MPs.

SEM images of HES MPs are shown in Figure 5. The MPs are semi-spherical in shape having a rough surface. No obvious change in the morphology of the MPs is observed before and after the drug loading process. The EDS analysis shows that the mass percentages of carbon and oxygen in KP are 79.28% and 20.72%, respectively (Figure 6). OFLX has 63.11% carbon, 5.78% nitrogen, 11.83% oxygen, and 19.28% fluorine by mass. In HES MPs, the mass percentages of carbon and oxygen are 48.63% and 51.37%, respectively. In HES-4 MPs, besides carbon and oxygen, nitrogen and fluorine are found to exist. This confirms the success in drug loading.

### 3.3. Evaluation of performance in drug encapsulation and release

**Figure 7.**
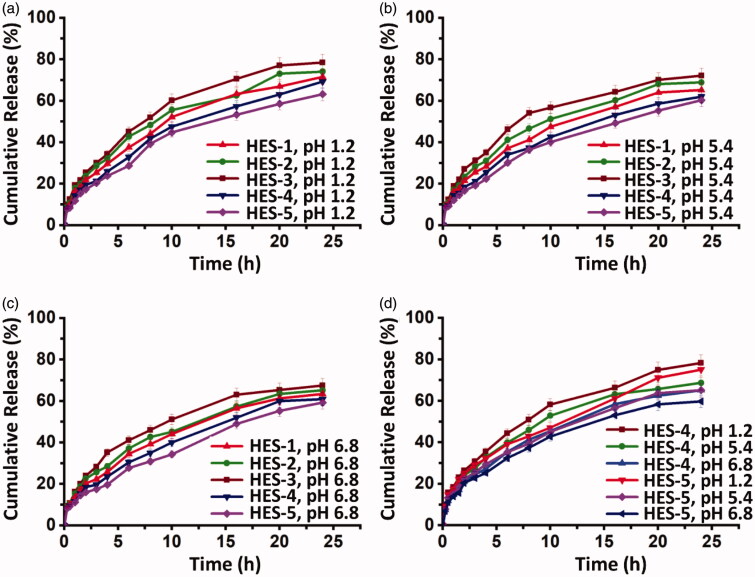
Profiles of release of (a, b, c) OFLX and (d) KP from different MPs at 37 °C under different pH conditions.

EE of the MPs ranges from 40.2 to 54.4%. It positively relates to the amount of the drug added during the process of drug loading, but negatively relates to the amount of the crosslinker added during MP fabrication. The latter is attributed to the fact that an increase in the amount of STPP increases the rigidity of the polymeric matrix, resulting in a decrease in the free volume space in the matrix for drug encapsulation. Apart from EE, profiles of drug release are studied. Based on the results (Figure 7), the rate of drug release decreases as the pH of the release medium increases. This is because the rate of hydrolysis of the ester bonds in MPs is higher when the pH decreases (Li et al., 2016). In PBS, OFLX and KP inside the MPs are not fully released. This is attributed to the stereo-protective effects of HES, which has a large molecular weight and a highly branched structure, to the loaded drugs, with the resistance of HES to hydrolysis also playing a role. A similar observation has been made in a study on drug release from the hydroxyethyl starch-10-hydroxy camptothecin conjugate (Li et al., 2016). In addition, by examining the release profiles of HES-1, HES-2, and HES-3 MPs, an increase in the drug content of the MPs is found to lead to an increase in the drug release rate regardless of the pH of the release medium. This is in good agreement with the observation made by Madhavi and coworkers (Chintha et al., 2020), who have studied the properties of sodium alginate/locust bean gum-*g*-methacrylic acid interpenetrating polymer network (IPN) hydrogels and have found that the rate of drug release from the hydrogels positively relates to the drug content.

**Figure 8.**
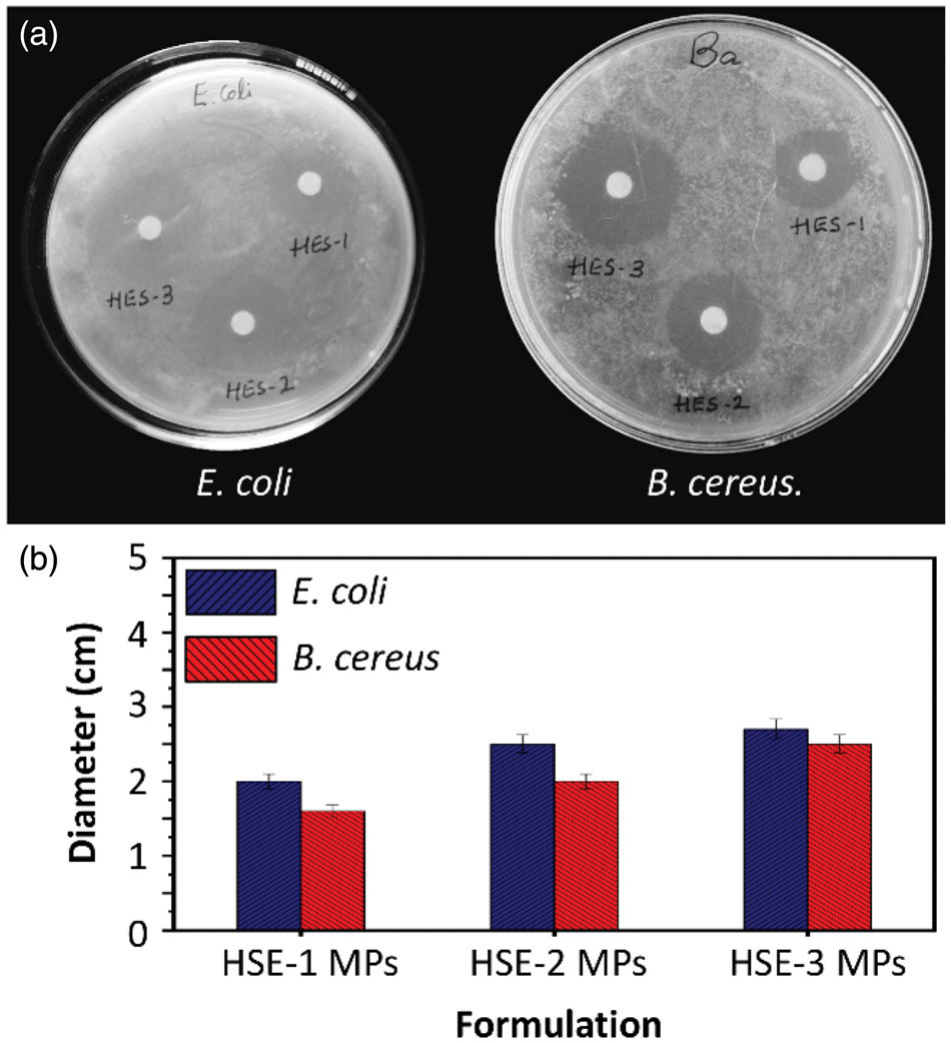
(a) Images showing the zone of inhibition induced by different MPs for *E. coli* and *B. cereus*. (b) The diameter of the zone of inhibition induced by different MPs.

The drug release data were fitted into different kinetic models (Table 2). Based on the correlation coefficients (*r^2^*) of the MPs, the profiles of drug release neither follow the zero-order model nor the first-order model. Instead, they fit the Higuchi model the most. This indicates that drug release from the MPs involves the diffusion of the release medium into the polymeric matrix, followed by diffusion of the drug molecules into the surrounding medium through the pores or intestinal channels of the MPs. The drug release data were fitted into the Korsmeyer–Peppas model using the following equation:

(2) $$\frac{M_{t}}{M_{\alpha }}=Kt^{n}$$

where *M_t_* is the mass percentage of the loaded drug released at time *t*, *M_α_* is the total amount of the drug loaded into the MPs, *k* is the release rate constant and *n* is the diffusion exponent indicating the type of drug release mechanisms. The *n* values are in the range of 0.415–0.549, indicating that the release process is mediated by Fickian diffusion. Finally, to demonstrate the capacity of the MPs in retaining the activity of the loaded drug, the antibacterial activity of OFLX-loaded MPs is examined by using *E. coli* and *B. cereus* as models (Figure 8). Because HES-3 MPs contain a high concentration of OFLX, they lead to the formation of the largest inhibition zone among formulations tested. This demonstrates that the MPs not only have high EE and drug release sustainability but can also retain the activity of the drug after the loading process.

**Table 2.** Release kinetic parameters of different formulations at pH 1.2, 5.4, and 6.8.

Drug	Formulation	pH	Zero-order		First-order		Higuchi		Korsmeyer–Peppas	
			*k*_0_	*r* ^2^	*k*_1_	*r* ^2^	*K_H_*	*r* ^2^	*n*	*r* ^2^
OFLX	HES-1 MPs	1.2	3.726	0.6360	0.069	0.8985	15.260	0.9935	0.488	0.9939
		5.4	3.459	0.5506	0.061	0.8307	14.259	0.9903	0.459	0.9952
		6.8	3.323	0.6272	0.056	0.8592	13.606	0.9940	0.484	0.9947
	HES-2 MPs	1.2	3.929	0.5676	0.079	0.8852	16.188	0.9883	0.465	0.9917
		5.4	3.700	0.5036	0.071	0.8356	15.319	0.9842	0.447	0.9928
		6.8	3.450	0.5377	0.061	0.8214	14.236	0.9901	0.455	0.9961
	HES-3 MPs	1.2	4.220	0.5531	0.092	0.9094	17.406	0.9867	0.462	0.9909
		5.4	3.951	0.3567	0.084	0.8058	16.534	0.9582	0.415	0.9835
		6.8	3.680	0.4431	0.071	0.8083	15.314	0.9747	0.433	0.9893
	HES-4 MPs	1.2	3.480	0.7127	0.060	0.9113	14.132	0.9937	0.518	0.9944
		5.4	3.199	0.6353	0.052	0.8522	13.086	0.9952	0.486	0.9957
		6.8	3.131	0.7134	0.050	0.8865	12.701	0.9932	0.518	0.9939
	HES-5 MPs	1.2	3.213	0.7177	0.052	0.8999	13.038	0.9900	0.523	0.9912
		5.4	3.027	0.6934	0.047	0.8712	12.308	0.9949	0.509	0.9951
		6.8	2.915	0.7728	0.044	0.8980	11.727	0.9843	0.549	0.9891
KP	HES-4 MPs	1.2	4.129	0.4870	0.088	0.8657	17.098	0.9849	0.441	0.9956
		5.4	3.707	0.4611	0.071	0.8174	15.392	0.9791	0.436	0.9920
		6.8	3.427	0.6007	0.060	0.8541	14.068	0.9939	0.474	0.9956
	HES-5 MPs	1.2	3.809	0.5647	0.072	0.8406	15.649	0.9903	0.459	0.9950
		5.4	3.421	0.5470	0.060	0.8160	14.092	0.9911	0.456	0.9967
		6.8	3.156	0.5867	0.052	0.8224	12.971	0.9925	0.470	0.9951

## 4. Conclusion

The development of carriers for concomitant administration of multiple bioactive agents shows high practical significance because it makes multi-drug therapy possible. Unfortunately, till now most of the reported carriers are designed for the delivery of single agents and hence fail to meet the need imposed by the co-delivery of multiple agents. In this study, we report the generation of MPs from HES as carriers of both KP and OFLX. Although only KP and OFLX are used to evaluate the performance of the MPs, because the process of drug loading is mediated simply by physical entrapment of drug molecules within the polymeric matrix of the MPs, it does not rely on the presence of specific functional groups on the drug molecules and hence is expected to be applied to drugs with diverse structures and properties. Along with the high drug release sustainability of the MPs and with the fact that the activity of the loaded drug can be maintained upon the drug loading process, our MPs show high potential to be further developed into carriers for applications in which co-administration of multiple bioactive agents is required.
